# Development and evaluation of a real-time PCR assay for the quantitative detection of *Theileria annulata* in cattle

**DOI:** 10.1186/1756-3305-5-171

**Published:** 2012-08-13

**Authors:** Amaia Ros-García, Antoni Nicolás, Ana L García-Pérez, Ramón A Juste, Ana Hurtado

**Affiliations:** 1 Department of Animal Health, NEIKER - Instituto Vasco de Investigación y Desarrollo Agrario, Berreaga 1, Derio, Bizkaia, 48160, Spain; 2 Menorca Gestió Veterinària, Occs Menorca, SL, S’Ull de Sol 1, Alaior, Menorca, Balearic Islands, 07730, Spain

**Keywords:** Theileria annulata, Mediterranean theileriosis, Quantitative real-time PCR, Spain, parasitaemia, Ticks

## Abstract

**Background:**

The tick-borne apicomplexan bovine parasite *Theileria annulata* is endemic in many tropical and temperate areas, including Minorca (Balearic Islands, Spain). Real-time PCR is widely used for the detection of piroplasms but quantification is not commonly considered.

**Results:**

We developed a real-time quantitative PCR (qPCR) assay for the detection and quantification of *T. annulata* that included an internal amplification control (IAC) to monitor for the presence of potential inhibitors. Specificity, sensitivity, precision, linear range and PCR efficiency were calculated and different methods for transformation of quantification cycle (Cq) values into quantities (Q) were evaluated. The assay was able to detect (100% probability) and quantify (linear response) 100 gene copies, and clinical sensitivity was set at 10 *T. annulata* per μl of blood. The assay was then validated on 141 bovine blood samples analyzed in parallel by a Luminex® suspension array, showing the utility of the qPCR assay developed here for the detection and quantification of the parasite in field conditions. Once validated it was used to monitor *T. annulata* parasitaemia throughout a year in 8 carrier animals from a farm in Minorca.

**Conclusions:**

The developed qPCR assay offers a reliable and simple way to quantify *T. annulata* infection loads, which could prove crucial in studying the role of carrier animals as a source of the infection, or assessing the efficacy of treatment and control measures.

## Background

Tropical theileriosis, also called Mediterranean theileriosis in the Mediterranean basin, is caused by infection with the protozoan parasite *Theileria annulata.* This disease is one of the most serious types of bovine theileriosis in Southern Europe, North Africa and Asia [1,2]. Main clinical signs associated with the infection include fever, swelling of superficial lymph nodes, inappetence, tachycardia, dyspnoea and weakness, and anaemia, leukopenia and lymphocytopenia are the most common haematological alterations in acute *T. annulata* infection. Decreased milk production may be observed in chronic infections in dairy cattle.

The parasite is transmitted by several species of ticks of the genus *Hyalomma*. In Spain, *T. annulata* is restricted to Central, Southern and Eastern parts of the country where the tick vectors *Hyalomma lusitanicum* and *Hyalomma marginatum* are present [3,4]. In Minorca, a small Mediterranean island in the Balearic Islands (Spain), Mediterranean theileriosis is endemic; prevalence of *T. annulata* in cattle is high [5,6] and *Hyalomma* ticks are abundant [7]. Animals with persistent infections by *Theileria* spp. become asymptomatic carriers, and when enzootic stability is broken due to physiological or management changes, deaths can happen. Buparvaquone, currently the most effective anti-theilerial drug for cattle, is not available in the European Union, and tick-control measures are the only tools used to combat the infection in Minorca. The main livestock species in the island is cattle, with sheep representing a very small proportion; wild ungulates are absent and rabbits are the main wildlife species.

PCR has been used for the sensitive and specific detection of *T. annulata*[8-10]. More recently, hybridization methods with high multiplexing capacity such as Reverse Line Blot (RLB) and Luminex® suspension arrays have been developed for the simultaneous detection and identification of the several *Babesia* and *Theileria* species including *T. annulata*[11,12]. However, none of them allow reliable quantification of the parasite. Here, we described a real-time PCR assay that can be used for both the detection and the quantification of *T. annulata*, we compared its performance on field samples with a Luminex® suspension array and used it to monitor *T. annulata* parasitaemia throughout a year in adult carriers from an infected cattle herd in Minorca.

## Methods

### Design of a *T. annulata*-specific quantitative real-time polymerase chain reaction (qPCR) assay

The 18S rRNA gene sequences of *T. annulata* and other piroplasm species available in GenBank were aligned and a pair of primers (Tann-18SF: 5’-AGACCTTAACCTGCTAAATAGG-3’; Tann-18SR: 5’-CATCACAGACCTGTTATTGC-3’), were designed to amplify a 120 bp fragment. A TaqMan® LNA™ hydrolysis probe specific for *T. annulata* was designed (FAM 5’-AAG[+T]TT[+C]TA[+C]TG[+T]CCCGTT-3’ BHQ1) and synthesized by Sigma (Sigma Aldrich, St. Louis, USA). The assay was developed for the co-amplification of *T. annulata* and an internal amplification control (IAC) in each reaction. The IAC consisted of a plasmid containing a 68 bp fragment of the 16S rRNA gene of the bacterium *Yersinia ruckeri* (ATCC 29473), which is the causative agent of enteric redmouth disease in salmonid fish species [13] and therefore not found naturally in bovine blood. Primers and probe used for *Y. ruckeri* amplification were as described by Lund *et al*. [14], with a small modification to the probe that included an additional T at the 5’ end (HEX 5’-TGCGAGTAACGTCAATGTTCAGTGC-3’ Iowa Black® FQ) as synthesized by IDT (Integrated DNA Technologies, Inc., IA, USA).

For real-time PCR assay optimization, recombinant plasmids containing the cloned PCR products of *T. annulata* and *Y. ruckeri* were generated using the appropriate primers for each target. The PCR products were purified and inserted into a PCR®4-TOPO® vector (TOPO TA Cloning® kit for Sequencing, Invitrogen, CA, USA) following manufacturer’s instructions. Cloned PCR products were sequenced using the ABI BigDye^TM^ Terminator Cycle Sequencing Ready Reaction Kit and an ABI3130 genetic analyzer (Applied Biosystems, Foster City, CA, USA). Sequences were subjected to BLAST search in the GenBank database and showed 100% homology with the target sequences. Plasmids were then linearized and spectrophotometrically quantified with a NanoDrop ND-1000 Spectrophotometer (NanoDrop Technologies Inc., Wilmington, DE). Copy numbers of the cloned 18S rRNA gene were derived from the molecular weights of the cloning vector and insert, and diluted in 10 mM Tris–HCl, pH 8.0 to generate standards ranging from 1 to 10^6^ molecules.

Real-time quantitative PCR was performed in MicroAmp^TM^ Fast optical 96-well reaction plates covered with thermo-sealing 4titude Clear Seal adhesives (Surrey, UK) at 180°C during 2 seconds in a 4s2™ Thermal Sealer (4titude Ltd., Surrey, UK). Analyses were performed using an ABI PRISM 7500 Sequence Detection System (Applied Biosystems) in 20 μl volume reactions. Optimized conditions consisted of 1x EXPRESS qPCR SuperMix (Invitrogen), 50 nM ROX Reference Dye, 0.2 μM of each primer, 150 nM of *T. annulata* probe, 200 nM of the IAC *Y. ruckeri* probe, 5 μl of extracted DNA and 100 copies of the IAC plasmid (optimal IAC plasmid amount for detection without affecting *T. annulata* detection limit). PCR cycling conditions were 50°C for 2 min, followed by 95°C for 10 min and then 40 cycles of 95°C for 15 s and 60°C for 1 min. Samples were analysed in duplicate along with the extraction negative controls and, at least three non-template negative controls were included in each plate. A 10-fold dilution series (10^6^ - 1 copy) of the plasmid DNA was analyzed in triplicate in each plate run.

### Real-time qPCR assay performance evaluation

The analytical specificity of the *T. annulata* real-time PCR assay was evaluated using plasmid DNAs containing inserts corresponding to the 18S rRNA gene of other piroplasms including the closely related species *Theileria parvaTheileria lestoquardiTheileria equiTheileria* sp. OT1, *Theileria* sp. OT3, *Babesia ovisBabesia motasiBabesia occultansBabesia bigemina* and *Babesia divergens* constructed as described elsewhere [15]. Blood from a known negative animal (not exposed to ticks) was also tested.

Ten-fold serial dilutions (10^6^ - 1 copy) of the linearized *T. annulata* recombinant plasmid were analyzed in 9 separate plate runs with three replicate reactions per dilution step. Results from these dilution series were used to assess the linearity and analytical sensitivity of the real-time PCR quantification protocol. The analytical sensitivity or limit of detection (LoD) of the assay, defined as the lowest concentration (target gene copy number per reaction) where ≥ 95% of test runs give positive results, was estimated using these serial dilutions. The limit of quantification (LoQ), defined as the minimum concentration (target gene copy number per reaction) that remained within the linear region of target concentration response, was also determined. Intra-assay and inter-assay reproducibility of the *T. annulata* TaqMan qPCR assay were represented as the standard deviation (SD) of the Cq values determined for the 10-fold serial dilutions of plasmid PCR standards analyzed.

The clinical sensitivity (or sample limit of detection) of the qPCR assay was determined using blood from an animal with clinical symptoms of Mediterranean theileriosis. Haematological examination revealed anaemia with low values in red blood cells (3.58 x 10^6^ erythrocytes/μl), low packed cell volume (23%) and leukopenia (2.3 x 10^3^ leukocytes/μl). Giemsa-stained slides were examined under oil immersion using × 100 objective lens and 51% of the erythrocytes were parasitized by piroplasms. The number of erythrocytes and intracellular forms with morphology compatible with *T. annulata* present in 20 microscopic fields were counted. Mean parasitaemia was set at 0.78 theilerias/erythrocyte and parasitaemia estimated at 2.79 x 10^9^ theilerias/ml. Blood was serially diluted (1/10, from 2.79 x 10^9^ to 2.79 x 10^4^ parasites/ml and 1/2 thereafter, to 50 parasites/ml) and dilutions were processed as described above.

### Real-time qPCR data analyses

Raw data from qPCR were recorded by the detection software SDS (v. 2.0.5, Applied Biosystems). The automatic settings for baseline and cycle threshold value were checked and adjustments made if necessary prior to calculation of the quantification cycle (Cq) values.

Three methods were compared for the transformation of Cq values into an estimate of copy number (Q) per reaction tube: i) the “single” approach, using the standard curve included in each run; ii) the “master” approach, using the curve obtained by the linear regression equation calculated using all Cq values (from triplicates) obtained for each standard dilution (27 Cq measurements), and; iii) the “mixed approach” which is a hybrid of the ‘master’ and ‘single’ strategies where the slope is fixed based on a series of repeated instrument runs, but the intercept is allowed to vary from run-to-run [16]. Twenty-five blood samples were analyzed in two different plates, and Q values obtained with the three calibration curve strategies compared.

To plot a mean standard curve (“master” curve) and calculate the standard linear regression equation using SigmaPlot^®^ (version 10.0), all Cq values obtained for the standard dilutions (9 runs, 27 Cq measurements) were considered. A calibration curve was fitted to these data by plotting the mean Cq values as a function of the known starting concentration of the standard dilutions. The standard linear regression equation thus calculated was then used to transform qPCR raw data from Cq values to an estimate of copy number (Q) per reaction tube (“master” approach). The slope of the master curve was used in the mixed approach along with the intercept of the single curve included in each run.

Once Cq values were transformed into an estimate of copy number (Q) per reaction tube using either of the above mentioned methods, the level of parasitaemia (P), expressed as number of *T. annulata* cells per ml of blood, was calculated by multiplying each sample Q value by 50 in order to account for the dilutions and volume transformations during sample processing, and the target gene copy number. The calculations were as follows:

(1) $$\text{P}=\text{Q x}\;\left({\text{V}}_{\text{B}}/{\text{V}}_{\text{EX}}\right)\;\text{x}\;\left({\text{V}}_{\text{EL}}/{\text{V}}_{\text{T}}\right)\;\text{x}\;\left(1/\text{CN}\right)$$

where V is defined as volume in μl and represents the following: volume of blood to refer the results to, i.e., 1 ml of blood (V_B_ = 1000 μl), sample volume extracted (V_EX_ = 200 μl), nucleic acid extraction eluate (V_EL_ = 100 μl), and nucleic acid template added to the PCR reaction (V_T_ = 5 μl); and CN is the gene copy number (2 copies per genome).

Percentage of parasitaemia expressed as the number of parasites per 100 red blood cells (RBC), was calculated as the proportion between the number of *T. annulata* cells per ml of blood determined by qPCR and the total RBC count per ml of blood measured for each sample as described below.

### Field samples

To assess the diagnostic test performance, the assay was then validated on cattle blood samples collected in two regions of Spain, one where Mediterranean theileriosis is endemic (Minorca, Balearic Islands, 102 samples from 2 farms) and another where the tick vector is not present (Basque Country, northern Spain, 39 samples from 3 farms). Forty-five blood samples from Minorca, which had been collected every 2 months throughout a year from 8 animals aged between 2–10 years, were then used to monitor *T. annulata* parasitaemia.

Leukocyte (WBC) and erythrocyte (RBC) counting was carried out with an electronic counter (Iber-cell, Barcelona, Spain). Packed cell volume (PCV) was measured by the standard microhaematocrit method, and haemoglobin concentration was determined by colorimetric methods. Leukocyte differential counting was carried out by thin blood smears stained with Giemsa and analysed under an oil-immersion objective, differentiating at least 100 cells.

### DNA extraction

DNA was extracted from 200 μl of blood using the BioSprint 96 DNA Blood Kit and the BioSprint 96 Workstation (Qiagen, Hilden, Germany). Negative controls were included every 10 samples to monitor for possible contamination. Extracted DNA was re-suspended in 100 μl of elution buffer and stored at −20°C until subsequent analysis.

### Molecular detection and identification of piroplasms by Luminex xMAP technology

PCR amplicons of the hypervariable V4 region of the 18S rRNA gene were generated as previously described [17] and identified using the Luminex xMAP technology and species-specific probes for bovine piroplasms [12]. The median fluorescent intensity (MFI) of each reaction was measured in a Luminex 200® system (Austin, TX) at 54°C. Positive/negative cut-off values were calculated for each assay and probe as described elsewhere [12].

### Statistical analyses

For statistical analyses, parasitaemia values were transformed into logarithmic values (log_2_) of the number of *T. annulata* cells per ml of blood. Correlation analyses were performed to search for any associations between haematimetric variables and parasitaemia data. Then, differences on average parasitaemia values regarding age [categorical; heifers (<3 yr), cows (≥3 yr)] and month of sampling, were assessed by means of GLM procedure and comparison of means. SAS statistical package version 9.1 (SAS Institute Inc., Cary, NC, USA) was used for statistical purposes. *P* values less than 0.05 were considered significant.

The level of agreement between methods (Luminex and qPCR performed in parallel on the same samples) was tested by the Kappa (K) index test at a 95% confidence interval using Win Episcope 2.0.

### Ethical considerations

Samples were collected by clinical veterinarians as part of the usual screening scheme on farms and Spanish ethical guidelines and animal welfare regulations (RD 1201/2005) were strictly respected. All herd owners had given an informed consent prior to the study.

## Results

### *T. annulata* qPCR optimization and data analysis

The developed qPCR assay amplified a 120 bp fragment of the 18S rRNA gene of *T. annulata* and a 68 bp fragment of the 16S rRNA gene of the bacterium *Y. ruckeri* from recombinant plasmids containing their corresponding cloned PCR products. No amplification signals were detected for other *Babesia* and *Theileria* species like *T. parva*, *T. lestoquardi*, *T. equi*, *Theileria* sp. OT1, *Theileria* sp. OT3, *B. ovis*, *B. motasi*, *B. occultans*, *B. bigemina* and *B. divergens*, or from the negative controls. When Cq values for the IAC were undetermined or above 34 in the absence of amplification signal for the *T. annulata* target DNA, the qPCR was considered inhibited and the reaction repeated.

**Figure 1:**
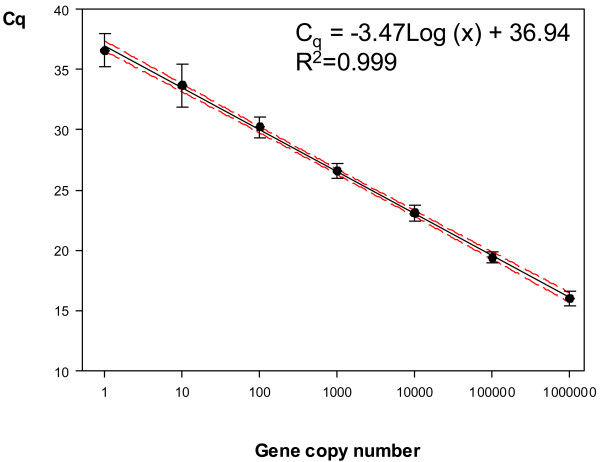
**Master curve generated by plotting the mean Cq values as a function of the known starting concentration of the standard dilutions.** Ten-fold serial dilutions (10^6^ - 1 copy) of the linearized *T. annulata* recombinant plasmid were analyzed in 9 independent experiments with three replicate reactions per dilution step. Error bars represent standard deviations (SD) of Cq values from replicated standards. Confidence intervals (CI) of the means of Cq values from replicated standards are indicated by the dashed lines.

The unknown samples that were analyzed twice in two different plates had Cq values ranging from 19 to 32 and showed good reproducibility, with very small variation in Cq results between runs (coefficient of variation, CV < 3.5%). However, the transformation of Cq values into an estimate of copy number per reaction tube (log_2_ Q) using the “single” model showed important differences in Q values between runs (CV_log2Q_ = 2.7-26%). The “mixed” approach showed similar or even higher differences (CV_log2Q_ = 7.3-37.4%). Finally, the “master” model reduced the CV_log2Q_ to 0.1-7.1% with values below 3% in 17 replicates. Therefore, the master standard linear regression equation calculated as described above was used to transform qPCR raw data from Cq values to an estimate of copy number (Q) per reaction tube. The standard curve thus obtained (y = −3.47Log(x) + 36.94; Figure 1) showed good linear response and a linear detection over at least seven log units, with a regression coefficient (R^2^) of 0.999 and an efficiency of 94%. The analytical sensitivity of the assay was 100% at the range of 10^6^ to 100 copies of the target gene in the qPCR assay, but decreased to 88.8% at 10 copies and to 55.5% at 1 copy. Therefore, the analytical LoD, defined as the concentration at which 95% of positive samples are detected, was set at 100 copies. The analytical LoQ was also set at 100 copies corresponding to a mean Cq of 30.22 (95% CI: 30.39-30.05).

The clinical sensitivity was determined by analysing serial dilutions (in triplicates) of infected blood from an animal with a known level of *T. annulata* parasitaemia. The lowest dilution that produced amplification corresponded to 220 *T. annulata*/ml of blood (6 x 10^-5^% parasitaemia). However, only for dilutions ranging from 2.79 x 10^8^ to 870 *T. annulata*/ml were all 3 replicates positive; 2 further dilutions gave positive signals in 2 replicates (440 *T. annulata*/ml) or 1 replicate (220 *T. annulata*/ml). Thus, clinical LoD was set at 870 *T. annulata* per ml of blood, which corresponded to 2.4 x 10^-4^% parasitaemia. Transformation of Cq values obtained from the dilutions into number of parasites per ml of blood showed good correspondence with values determined by microscopy. For example, the dilution that produced an amplification at the established analytical LoQ (Cq ~ 30.22), contained 6975 parasites/ml of blood according to microscopy and 5620 *T. annulata*/ml as estimated by qPCR. These results would demonstrate reliable quantification of *T. annulata* (clinical LoQ) in blood field samples from as little as 10^4^ *T. annulata* per ml of blood (10 parasites per μl).

### *T. annulata* qPCR performance compared with Luminex

The performance characteristics of the qPCR assay were assessed by testing in parallel by qPCR and Luminex 141 cattle blood samples. All samples collected in the Basque Country (39) were negative by both techniques. Positive samples were only detected in Minorca. The results of real-time qPCR assay were compared with those of a Luminex® suspension array showing a very good kappa index of agreement (Kappa = 0.97). Only one of the 66 samples positive by Luminex was negative by qPCR, whereas another sample negative by Luminex was positive by qPCR. However, estimated values for 3 samples (one of them corresponding to a Luminex negative sample) were below the LoQ so that quantitative data would be less reliable (Q = 234, 589 and 840 *T. annulata*/ml blood). Most *T. annulata* positive samples had been identified as mixed infections with either *T. buffeli* (21) or *B. major* (28) by Luminex, but this did not affect qPCR sensitivity.

### A year-long monitoring of *Theileria annulata* infection

**Figure 2:**
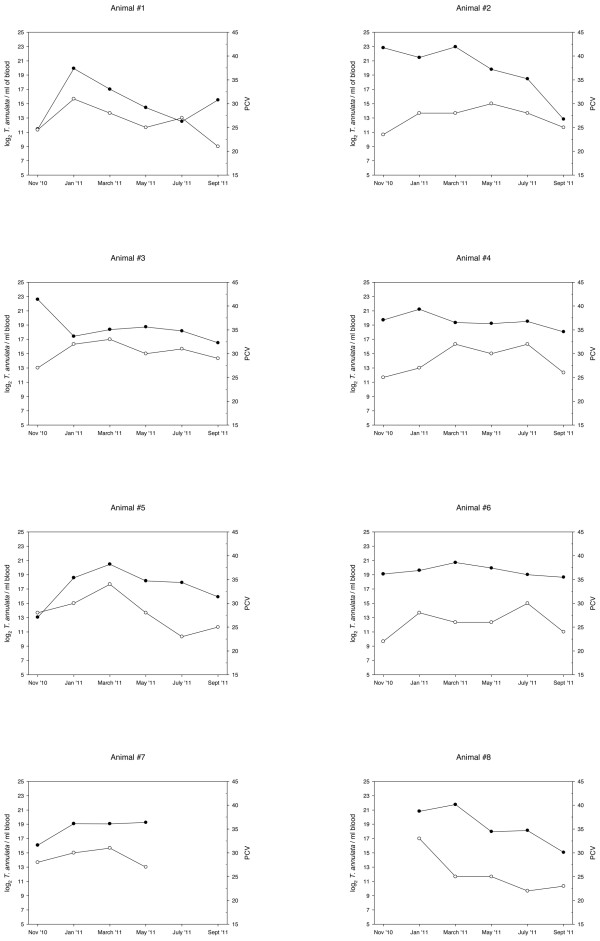
**Graphical representation of individual parasitaemia (log**_**2**_** *T. annulata* /ml of blood) variations (black dots) throughout the study along with the corresponding PCV results (open dots) for each animal.** Age of animals (in months) at first sampling was as follows: Animal #1, 92; Animal #2, 49; Animal #3, 79; Animal #4, 37; Animal #5, 47; Animal #6, 46; Animal #7, 121; Animal #8, 26.

**Figure 3:**
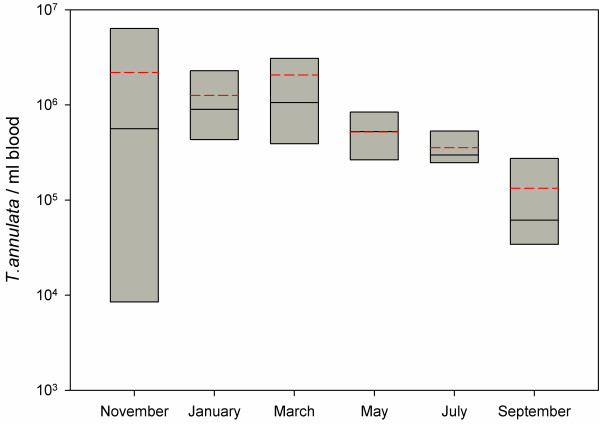
**Box Plot representing *T. annulata* per ml of blood in the animals grouped according to the sampling time.** The boundary of the box closest to zero indicates the 25th percentile, the continuous line within the box marks the median, the dashed line marks the mean and the boundary of the box farthest from zero indicates the 75th percentile.

Fluctuations on parasitaemia levels could be monitored throughout a year in 8 carrier animals that remained positive during the study period in one of the farms from Minorca. The haematological parameters (PCV, haemoglobin, and RBC) of these animals remained close and occasionally below the lowest values considered normal for cattle, but no correlation was found between parasite load and haematology values. Estimated parasitaemia loads ranged between 2.75 x 10^3^ and 8.14 x 10^6^ *T. annulata*/ml blood (corresponding to 5 x 10^-5^ to 1.5 x 10^-1^% *T. annulata* per 100 RBC), being the observed mean value of 1.1 x 10^6^ *T. annulata*/ml blood. Individual parasitaemia variations throughout the study are shown in Figure 2 along with the corresponding PCV values. At the beginning of the study, in November, differences in parasite loads among the different animals were the largest and the mean parasitaemia the highest (Figure 3). In most of the animals the peak of maximum parasitaemia was observed either in January or March, the exception being animal #3 which showed a peak at the beginning of the study that then dropped and remained at *ca.* 10^5^ *T. annulata*/ml blood. In May onwards, *T. annulata* infection rate decreased and at the end of the study, in September, parasitaemia had the lowest mean values, and differences were significant (P < 0.05) when compared with mean values observed in January and March. No differences were found between heifers and adult animals when comparing parasitaemia data for each sampling date.

## Discussion

Several molecular tests based on PCR and hybridisation assays (RLB or Luminex®) are available for the detection and identification of *T. annulata*[9-12]. However, none of them allow reliable quantification of the parasite, which could prove very useful in following response to antitheilerial treatments, monitoring experimental infections or studying the role of carrier animals as a source of infection. Here, we described a real-time PCR assay that can be used for both the detection and the quantification of *T. annulata*, and used it to monitor *T. annulata* parasitaemia in carrier cattle. Real-time quantitative PCR has a great potential for analytical applications in quantitative DNA analysis, but providing quantitative data requires a more complicated data analysis than qualitative PCR. Several methods can be used for transformation of qPCR raw data from a Cq to an estimate of concentration from an unknown sample and there is not clear consensus on the best approach. Here, we ran 9 independent standard curves, 5 of them along with the unknown blood samples. In total, 27 replicates of the 7 ten-fold plasmid serial dilutions (10^6^ - 1 copy) were amplified and, results from these dilution series were used to plot a mean standard curve (“master” curve) and to calculate the standard linear regression equation. The inclusion of an independent standard curve accompanying each experiment and the repetition of unknown samples in different runs allowed the comparison of different methods for the transformation of Cq values into Q estimates. Results showed that the use of the mean standard curve better controlled experiment-to-experiment variability between plasmid replicate measurements associated to variation in dilution preparation and storage and provided better reproducibility of sample quantitative data estimates. However, the use of a master curve does not preclude the inclusion in each run of positive controls, ideally at both limits of the dynamic range, which should fall within the master curve values for assay validation.

The newly developed qPCR assay showed high specificity and sensitivity and, reliable and reproducible quantification over a broad range of template concentrations. The sensitivity and linear detection range were found to be high and broad enough to reliably detect and quantify as little as 100 target gene copies per reaction tube. Methodological variation was determined to be well-controlled within PCR runs and albeit slightly higher among replicate runs, reproducibility was good.

Here, we assumed that there are no differences between respective genomic and plasmid DNA types, which does not always hold true [18]. In an attempt to validate findings from real-time PCR quantification on true biological samples, qPCR results were compared with those obtained from more traditional approaches like microscopy counts using serial dilutions of blood from an infected animal. Genetic and microscopy methods measure different units (gene copy number *vs.* infected cells) and therefore, transformations are needed before comparison is possible. A simple transformation as described here, allowed comparison and showed good correlation of quantitative data generated by real-time PCR quantification and the number of parasites estimated by microscopy. The diagnostic test performance of the qPCR assay was also validated by the analysis in parallel of clinical bovine blood samples using a Luminex® suspension array, and concordance between techniques was shown to be very good. However, clinical sensitivity of qPCR (10 *T. annulata*/μl blood) was lower than that of Luminex®, previously reported to be 0.05 *T. annulata*/μl [12]. Nevertheless, the results presented herein demonstrated the suitability of the qPCR assay for reliable quantification of *T. annulata* in blood samples from carrier animals with parasitaemias as low as 5 x 10^-5^%. LoD was comparable to that determined for other real-time PCR procedures designed to detect different piroplasm species, which have been reported to range between 1.1 x 10^-4^% to 1.5 x 10^-5^% parasitaemia [19-21].

Following international standards for PCR-based diagnostic techniques [22], an IAC was added to each real-time PCR reaction and used as an indicator of potential PCR reaction inhibition. Since partial inhibitions can cause an infra-estimation of the actual number of target gene copies, a Cq of 34 was set as the validation limit for IAC amplification. Presence of PCR inhibitors could thus be monitored and associated errors excluded.

Once validated, the qPCR assay was used to monitor *T. annulata* infection dynamics throughout a year in 8 naturally infected animals from a herd in a region where *T. annulata* is endemic. Parasitaemia levels among these animals (mean 1.1 x 10^6^ *T. annulata*/ml blood) were clearly below those detected in the animal with clinical symptoms of Mediterranean theileriosis used to calculate the clinical sensitivity of the assay (2.79 x 10^9^ *T. annulata*/ml blood). Thus, observed values were indicative of subclinical infections and consistent with the status of asymptomatic carriers. A peak was observed in January – March, but could not be associated with any changes in management or production system within the herd. The degree of parasitaemia of carrier animals may affect the level of infection acquired by feeding ticks. An overlap between high parasitaemia levels and a peak of activity of *Hyalomma* vector ticks could pose a higher risk for transmission. Presence of *Hyalomma* ticks feeding on animals from this farm has been recorded (data not shown), but data on tick population seasonality in Minorca are scarce. In the only study on cattle ticks carried out in Minorca, *Hyalomma* ticks were collected from spring to autumn, with the majority of *H. marginatum* adults being recorded in spring-summer and *H. lusitanicum* being more common in autumn (Castellá *et al.* 2001).

Although haematological parameters of these animals remained close and occasionally below the lowest values considered normal for cattle, no correlation was found between parasitaemia and haematology values. Major haematological alterations would be expected in young naive animals when first exposed to infected ticks. Unfortunately, calves were not sampled in this study; the sampled animals (heifers and cows) were positive to *T. annulata* from the beginning of the study. In fact, the year before this study started, 4 deaths had been recorded in the farm (one 7 month-old calf and 3 cows), but no laboratory analyses were performed at the time to identify the cause. It would be interesting to monitor the initial stages of the infection when calves start getting exposed to *T. annulata* once maternal antibodies wane. This would allow investigating the dynamics of *T. annulata* infection from the initial stages of parasitaemia, its pathogenic effect after massive replication of the parasite inside erythrocytes and leukocytes and its impact in haematological parameters, which could not be clearly observed in this study.

## Conclusions

This study described a TaqMan LNA^TM^ real-time PCR assay targeting the 18S rRNA gene that allowed sensitive detection and quantification of *T. annulata*. The minimum information necessary for evaluating qPCR experiments according to MIQE guidelines [23] was provided for the procedure design, qPCR validation and data analysis, and its utility in field conditions was assessed on cattle blood samples. This qPCR assay constitutes a valuable addition to the repertoire of parasite detection tools currently available for studies of *T. annulata*, and the quantitative data that the technique provides should contribute to enhanced understanding of *T. annulata* infections.

## Competing interests

The authors declare that they have no competing interests.

## Authors’ contributions

AR carried out the experimental work and participated in drafting of the manuscript. AN collaborated in the design of the field study and carried out sample collection. ALG performed the statistical analysis, interpreted data and critically revised the manuscript. RAJ participated in the critical reading of the publication. AH conceived the study, participated in its design and coordination, and drafted the final manuscript. All authors read and approved the final manuscript.
